# A Review of Risk Factors for Polypharmacy: Age, Level of Education, and Physician's Attitude

**DOI:** 10.7759/cureus.71868

**Published:** 2024-10-19

**Authors:** Gabriel Majewski, Olga Grodzka, Radosław Walkowski, Tomasz Kandefer, Kinga Papciak, Stanisław Słyk, Izabela Domitrz

**Affiliations:** 1 Department of Neurology, Medical University of Warsaw, Warsaw, POL

**Keywords:** economical aspects of health, neurology, patient-doctor relation, polypharmacy, public health education

## Abstract

Polypharmacy, a growing public health and economic concern, is particularly common among the elderly due to the high prevalence of multimorbidity, such as dementia and stroke, which necessitates complex treatment regimens. While commonly understood as taking five or more medications, definitions of polypharmacy are varied and may be misleading in clinical practice. This research examines factors such as a country's expenditure on health and education, age, and clinicians’ holistic approaches to compare the prevalence of polypharmacy across different groups. The review included documentary research through PubMed, Scopus, and Google Scholar databases and search engines, resulting in seven selected sources. The average year of publication was 2020 (median: 2020; standard deviation: 1.63; range: 2018 to 2023). The level of polypharmacy was found to be significantly dependent on per capita expenditure on education (R^2^ = -0.79; F(6) = -3.11; p = 0.02) and health (R^2^ = -0.76; F(6) = -2.88; p = 0.03). Countries with higher spending in these areas had a lower proportion of participants with polypharmacy. Additionally, patients’ quality of life (QoL) is closely tied to the amount of medication they consume, highlighting the need for physicians to avoid unnecessary prescriptions. Patients impacted by polypharmacy often lack knowledge about their diseases and medications, negatively affecting their QoL and compliance. To develop effective treatment plans and improve clinical practice, doctors should consider these risk factors, prioritize patient education, and utilize innovative technologies to support patients. While polypharmacy is sometimes unavoidable and necessary, this approach could in some cases help mitigate the challenges and risks posed by inappropriate polypharmacy and enhance patients' QoL. Furthermore, policymakers should consider increasing spending on education and healthcare, as this may resolve clinical and economic problems related to the issue.

## Introduction and background

Nowadays, holistic care for people is becoming more complex and challenging. The presence of numerous persistent diseases increases the complexity of treatment and health management for both doctors and patients. It is called multimorbidity and has been universally defined as the simultaneous suffering of two or more chronic health conditions (especially in neurological patients) in contrast to polypharmacy, whose definitions vary depending on source and context [1-3]. Multimorbidity was shown to appear more frequently in the elderly population [4], causing patients to spend more funds than ever in history [5].

Polypharmacy is a major and constantly growing public health issue occurring in all healthcare settings worldwide [6]. It appears to be particularly common in elderly patients [7], due to the high prevalence of multimorbidity, which necessitates complex treatment regimens for managing common chronic conditions such as dementia, stroke, hypertension, and diabetes. Older age is associated with differences in pharmacokinetic and pharmacodynamic characteristics compared to younger patients [7], which may cause medication-related problems, such as medication nonadherence. It can be linked to 125000 deaths in the U.S. annually, resulting in approximately 177 billion dollars in increased healthcare costs [7]. A systematic review and meta-analysis regarding the association between polypharmacy and death conducted by Leelakanok et al. indicates that mortality rises progressively with an increasing number of medications taken [8].

As it was mentioned above, there is no consensus on what should be understood as polypharmacy itself [9]. The most commonly used term for polypharmacy in modern literature is its numerical definition as five or more medications [6,9,10]. In other sources, it ranges from 2 to even 11 or more drugs [10]. Unfortunately, it poses a potential complication of direct study comparison in our review. However, numbers are not the only important feature to focus on. Thus, descriptive definitions were created [9]. The numerical ones have limited value in practice, and clinical aspects of treatment create the need to establish a term for appropriate (otherwise called rational) polypharmacy to distinguish between describing unnecessary medications and multidrug therapy [10]. It is important to identify those patients who may be at risk of endangered health outcomes as a result of inappropriate polypharmacy and indiscriminate prescribing. In our study, to keep the paper clear, the most common definition of polypharmacy, which is taking five or more drugs, has been used. In the case of other term interpretations in particular studies, the definition will be specified.

While in many cases using multiple drugs may be clinically appropriate, it is important to understand when the inappropriate one may harm patients with poor treatment outcomes and drug interactions, increasing side effects, toxicity, nonadherence, delirium, and fall risk [11]. Additionally, polypharmacy was shown to increase space for medical errors, reduce physician productivity, and overburden national medical systems [11]. This study aimed to evaluate the prevalence of polypharmacy in various groups and specify the relationship between selected features. Thus, the study divided the features into two subgroups: (i) patient-dependent factors and (ii) doctor-dependent factors. First, we focused on patient and country levels of education and age; subsequently, on dementia and the clinician’s holistic approach to compare the presence of polypharmacy among the discussed groups. Also, the collaboration allowed for the specification of factors that influence a patient's awareness of their diagnosis and therapy plan, which may be the main factor in deciding on present or absent polypharmacy.

## Review

Searching strategy

Study Design

The paper was written as a narrative review, although a systematic manner was partially implemented. An electronic article search included the following databases together with search engines: PubMed Database, Scopus Database, and Google Scholar as they remain an optimal tool in biomedical electronic research [12]. The search strategy for searching databases was as follows: (polypharmacy) ∧ (patient age ∨ level of education ∨ knowledge of a disease ∨ relationship of patient and doctor ∨ problem in individual countries). Then, Google Scholar was searched with the following keywords: “the structure of national budgets”; “the programme for international student assessment score (PISA)”; “PISA score”; “education and expenditure per capita,” as it returned more accurate results during the retrieval of even the most oblique information [12]. The literature research was conducted independently by two co-authors to avoid bias.

To answer whether the increasing expenditure on education and health care correlates with polypharmacy, we selected a limited number of data sources. They were searched through the previous databases under the keyword “Polypharmacy Prevalence (Country Name) of 65 Years.” The papers closest to each other in terms of year of publication were selected and duplicates were removed. The following formula was used to calculate “Expenditure on education per capita” as a fraction of gross domestic product (GDP) per capita.

\[ \frac{\text{GDP per capita} \times \text{Government expenditure on education (% of GDP)}}{\text{100}} \]

Inclusion and Exclusion Criteria

Only the high-quality articles mainly from impacted journals were included in the analysis. The first category of studies compared the issue of polypharmacy between predetermined age and educational levels; these studies were subsequently referred to as "patient-dependent factors." The second set of papers addressed a patient-centered approach, including "the patient-doctor relationship" and "the patient's knowledge of his disease," which are referred to as "doctor-dependent factors" from now on.

The case reports, conference abstracts, letters to the editors, and commentaries were not included. With some exceptions to demonstrate differences [13-21], we did not include publications that were released prior to 2010 to maintain the best possible convergence of data on the budget structure of the selected countries. Countries that didn’t have a minimum of two PISA-conducted exams and national budget data were rejected. The studies written in languages other than English were not taken into consideration.

Statistical Analysis

The obtained results were checked for normal distribution. For this purpose, the Shapiro-Wilk test and, thereafter, Spearman's rank correlation coefficient were performed. The significance level was set at p < 0.05. Microsoft Excel 365 (Microsoft Corp., Redmond, USA) was used to manage the collected data and Statistica 13.3 (Quest Software Inc., Aliso Viejo, USA) conducted statistical analysis. The G*Power 3.1.9.7 program (Heinrich-Heine-Universität Düsseldorf, Düsseldorf, Germany) was used to acquire the power of the tests used. The calculation was done post hoc to determine whether the data collected were of sufficient quality to be used further. The minimal acceptable power of the test (1-β error probability) was set at 0.8 [22].

Patient-dependent factors

Age

One of the greatest risk factors for polypharmacy is age [9,23]. Its increase is associated with the development and worsening of geriatric syndromes such as cognitive impairment, delirium, falls, urinary incontinence, and many other syndromes and conditions that require even multiple medications for treatment [24,25]. Moreover, the aging population is a cause of rising multimorbidity [9,23]. Recently, polypharmacy across the elderly population has become a global problem that is constantly getting worse [24].

A study conducted across 17 European countries and Israel examined the incidence of polypharmacy in three different age groups. The study proved a trend that the prevalence of polypharmacy increases with the age of the study participants. The lowest rate was found in the age group between 65 and 74, amounting to 25.3%. Furthermore, the rate for participants aged 75-84 was at 36.4%, and for participants aged 85 years or older, it was at 46.5% [26]. The association between polypharmacy and the elderly age of the patient was also highlighted in a systematic review. Two different age groups were included among the participants: (i) under 65 years and (ii) 65 years or older, whereas the age of participants ranged from 26 to 87 years old. Unsurprisingly, the prevalence of polypharmacy was higher for participants aged 65 years or older compared to participants under 65 years [27]. The coexistence of multiple health conditions is a common phenomenon in elderly people [28,29], which may be reflected in the number of medications taken. With reference to the number of drugs taken, two groups: polypharmacy and excessive polypharmacy were outlined in the cross-sectional analysis of a population-based cohort in Finland. Excessive polypharmacy was defined as taking 10 or more drugs and polypharmacy as the use of six to nine drugs. Interestingly, the correlation between participants aged 85 years or older and polypharmacy was not found, which may be a subject for further studies. However, this study revealed an association between age 85 years or older and excessive polypharmacy [18].

A general trend of increasing prevalence of polypharmacy with increasing age was also present in the study conducted in Funen, the third largest island of Denmark. However, in that study, the rate of polypharmacy was lower for individuals aged 90 years or older than for participants aged 80-89 years [14]. It could be explained that people who manage to live to 90 years or more are generally healthy, and usually, they are not affected by many chronic diseases; thus, there is no need for them to take a large number of medications. Nevertheless, studies to understand this issue better are highly required.

The prevalence of multimorbidity is closely linked to age, with a notable trend showing that polypharmacy increases as individuals grow older [9,26,27]. However, an interesting correlation emerges among those aged 90 years and older, where the prevalence of polypharmacy is actually lower than that of younger participants [14,18]. This may be attributed to the fact that many individuals who reach 90 tend to experience fewer health conditions and generally maintain better health, resulting in less need for multiple medications.

Selected Case: Polypharmacy in Patients Suffering From Dementia

Undoubtedly, polypharmacy is widespread among elderly patients, with the risk increasing with age [30]. This issue has already been analyzed in the previous part of this article; however, here appears another problem closely related to the former. Unquestionably, the prevalence of dementia is an important issue, especially among older adults, as it is well-known to rise with age and patients with dementia have the highest degrees of clusters of chronic morbidities (more than 50% of them with more than five chronic disorders), compared to other neurological disorders [1]. Therefore, the question is how dementia is correlated with polypharmacy.

Multiple studies have been conducted so far to show the correlation between the presence of polypharmacy and suffering from dementia. In a population-based case-control study in Taiwan [31], the patients with cognitive impairment were investigated and compared to the control group without dementia diagnosed. The use of medicines in the study group was significantly higher. Furthermore, the risk of dementia increased with the number of used medications. These results were confirmed by further studies conducted by other researchers [32,33], who observed a strong association of polypharmacy with cognitive decline. Both groups analyzed the patients suffering from dementia in the course of Parkinson’s disease (PD). Consistently, in another study [34] a higher frequency of polypharmacy (determined as taking over three chronic drugs) in patients affected by Alzheimer’s disease (AD) or PD was demonstrated compared to patients without cognitive impairments. Finally, another research group [35] concluded that the presence of PD, including patients with PD followed by mild cognitive impairments, increased the risk of polypharmacy. However, the study conducted by other researchers [36] showed no differences between the use of medications in patients with dementia when comparing fully dependent ones to those who were mobile and, at most, partially dependent. This research assessed polypharmacy based on two definitions: (i) using more than five drugs and (ii) using more than seven drugs.

The correlation between polypharmacy and dementia has been proven clearly. However, a particularly important matter that needs to be raised is the influence of polypharmacy on the quality of life (QoL) of patients suffering from dementia. In a cross-sectional study [37], the level of health-related QoL in the patients affected by AD was assessed. Polypharmacy (described here as three or more medications per day) was shown to be significantly related to lower QoL. Moreover, the correlation was proven according to the assessment of QoL by a caregiver and the patient’s self-report. Similarly, the influence of polypharmacy on self-reported QoL was presented in another study [38] where also patients suffering from AD were investigated. Another research group [16] focused on side effects caused by medications taken by elderly patients, the majority of whom presented the symptoms of dementia. It was demonstrated that the episodes of polypharmacy appeared commonly in the one-year assessment, resulting in the presence of side effects in the majority of patients. Interesting observations were made by other researchers [22], who conducted a study on patients suffering from advanced PD and dementia. In those cases, the withdrawal of dopaminergic medications to reduce polypharmacy did not cause significant motor deterioration compared to the maintenance of treatment. Surprisingly, cognition functions even improved in two patients after the withdrawal.

To summarize the role that polypharmacy plays in patients with dementia, the higher use of medications was shown not only to increase the risk of cognitive decline but also to worsen the QoL. Furthermore, the appropriateness of taking each of the medicines should be carefully assessed, as some of them may not provide benefits while the risk of causing side effects remains.

Level of Education (LoE)

Number of educational years spent in education: The importance of socioeconomic factors, mainly the number of educational years and LoE, was researched to find its impact on the prevalence of polypharmacy amongst examined patients.

The previously mentioned cross-European study [26] collected data from SHARE (Survey of Health, Ageing, and Retirement in Europe) across 17 different European countries and Israel. The survey was conducted across 68231 individuals aged between 24 and 106 years; however, researchers included individuals from the sixth wave of SHARE aged 65 years or more and taking at least five different drugs, giving a total of 34232 participants. Countries with the lowest prevalence of polypharmacy were Switzerland, Croatia, and Slovenia, while Portugal, Israel, and the Czech Republic had the highest prevalence of polypharmacy. Results showed a prevalence of polypharmacy ranged from 26.3% up to 39.9%. The information gathered showed that polypharmacy increased with a lower number of years of full-time education. Lower QoL, well-being, and higher network satisfaction were also found to be correlated. Interestingly, individuals who reported rarely, sometimes, and often shortage of money also showed a higher prevalence of polypharmacy [39].

A study among 1,705 elderly people living in Florianopolis, Brazil, showed the prevalence of polypharmacy to be 32%. When taking the number of education years into account, the prevalence of polypharmacy was, respectively, 34.4% in a group of 0-4 years spent in education, 33.3% in a group of 5-8 years, 34.2% in a group of 9-11, and 25.4% in a group of people that spent over 12 years during education. The authors noticed that the prevalence of polypharmacy was lower for individuals with a higher LoE - 12 or more years, whereas people with fewer years spent in education had a higher prevalence of polypharmacy [39].

Adherence to treatment is a critical factor in reducing the risk of polypharmacy and its associated dangers. A cross-sectional study conducted on 422 individuals in Brazil considered participants with either less than or more than eight years of education. Among the 268 participants with less than eight years of education, 126 did not adhere to their medication. Similarly, 54 out of 154 participants with more than eight years of education also failed to adhere to their treatment plans [40]. However, the study found no significant correlation between treatment adherence and LoE.

In contrast, another study conducted amongst 754 participants [41] expected that a low LoE would be associated with less medication knowledge. However, they found that LoE was statistically insignificant as a predictor of medication adherence/non-adherence. The group with “no or low” LoE correctly recalled the drugs’ indication in 15.3%, and intermediate or high correctly recalled the drugs’ indication in 13.1%. This topic will be further discussed in the doctor-dependent section of the article.

Non-adherence may be one of the most important negative aspects of polypharmacy that has a dire impact on the patient’s life; hence, a cross-sectional Chinese study [42] on a sample of 258 patients ≥80 years of age, at least one chronic disease, and taking at least five different types of medications to find the factors influencing medication compliance. Rates of non-adherence varied from 43% to 100% among different communities. The majority of patients (53.9%) had junior college diplomas, and 24.7% had a middle school education or lower. The average accuracy of individuals’ responses to the medication knowledge was 68.7%, and the highest average number of corrected responses referred to expired drugs, medication types, and drug storage. However, the areas with the lowest awareness were drug side effects, drug abuse, drug incompatibility, and dosage. Therefore, researchers found that medication knowledge declined with increasing age.

LoE depending on the country: LoE in different countries is definitely an overly complex topic. It is difficult to find one factor that is consistently relevant and differs from one country to another. This part of the study focused on comparing LoE by educational attainment [43-45], expenditure on secondary education [46], PISA 2018 study [47] (scores for the analyzed country summarized in Table 1) and checked the impact of it on polypharmacy levels.

**Table 1:** Summary of LoE in different countries N/A: not available; PISA: Programme for International Student Assessment; LoE: level of education Table adapted from the PISA 2018 study [47]. Data is a mean score, then divided by levels. Spain, Romania, and Jordan mostly found themselves on level 2, others on level 3.

Country	PISA 2018 reading	PISA 2018 mathematics	PISA 2018 science	Finished upper secondary	Bachelor's and doctor's
Sweden	506	502	499	77451	26371
USA	505	478	502	90941	39613
Spain	N/A	481	483	53257	24460
Belgium	493	508	499	71446	35907
Switzerland	484	515	495	86645	2963
Jordan	419	400	429	50244	23408
Poland	512	516	511	87778	28662
Romania	428	430	426	69182	13095

In many studies, the conclusions were remarkably similar. Lower education of patients means a higher probability of polypharmacy [17,48-54]. A study conducted in Sweden [17] showed that lower educational attainment was associated with a greater likelihood of polypharmacy, even after controlling for varied factors like age, although there seemed to be a difference between genders. Women with lower education had higher chances of polypharmacy compared to men with lower education. The explanation may be the difference in socioeconomic status between genders; however, more studies need to be conducted on that topic. Further studies, like the one in Spain, showed the same results on LoE [26]. Polypharmacy was proven to be associated with insufficient education. After ranking the countries in the studies used, there seemed not to be a significant impact of the LoE on overall polypharmacy levels.

The prevalence of polypharmacy can be affected by the LoE. A research team in Jordan investigated the prevalence of polypharmacy and its correlation with potential drug-drug interaction. The study found that over 74.9% of participants used five drugs or more. It was significantly higher than in Sweden [55], where the prevalence of polypharmacy was found to be 44%. Further studies conducted in different countries, like Poland, revealed that polypharmacy was present among 42.1% of participants aged 65 or more. Similarly, in the study carried out in the USA, the result was 36.8% among those above 65 years [56,57]. Many other factors, including socioeconomic status, comorbidities, and drug classification, may be the cause of this observation. Additionally, LoE may play a role as it reflects awareness toward drug use; nevertheless, more studies need to be conducted. The age difference of the participants may also influence the results and need to be evaluated.

Expenditure on Education and Health Care

For the purposes of our review, we carried out a correlation analysis of the ratio of people 65 and older with polypharmacy according to the expenditure of the country in which they live on education and health care.

**Figure 1:**
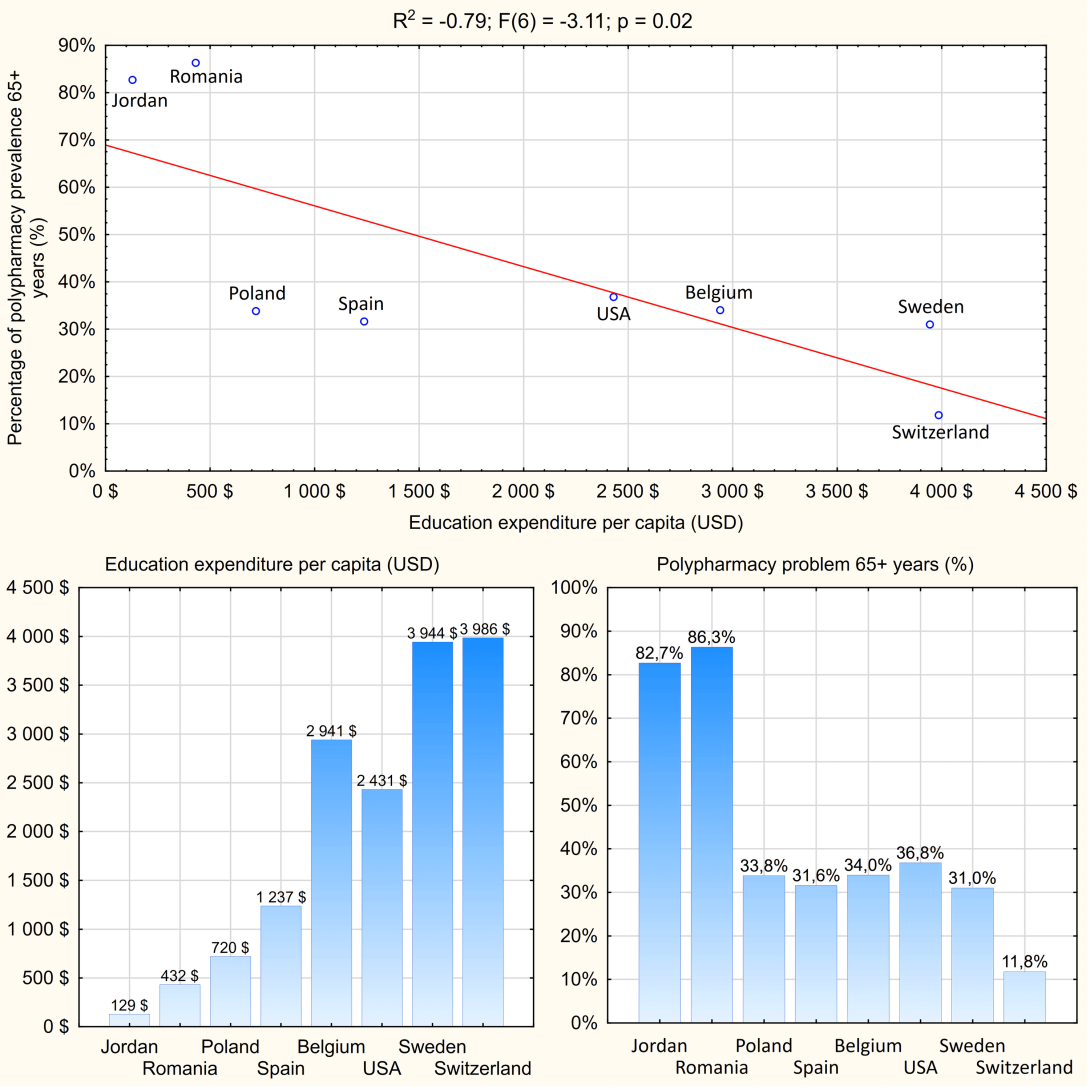
Correlation between education expenditure per capita (USD) and percentage of polypharmacy prevalence 65+ years Reference: [26,56,58-62] Credit: Image created by the author

A total of seven [26,56,58-62] sources were selected for the data presentation shown in Table 2. The countries that were indicated showed the typical highly and moderately developed European state structure. Moreover, one Middle Eastern country (Jordan) was considered to the contrary. The median year of publication was 2020 (SD (standard deviation) = 1.63; minimum = 2018; maximum = 2023). The distribution of countries' expenditure on education and health care followed a normal distribution (p = 0.27; p = 0.34), while the frequency of polypharmacy among people 65 and older did not (p = 0.02), as expected. The level of polypharmacy was found to be statistically significantly dependent on per capita expenditure on education (R^2^ = -0.79; F(6) = -3.11; p = 0.02) and health (R^2^ = -0.76; F(6) = -2.88; p = 0.03). In addition, both tests achieved the required >0.8 power of the test [22]. Countries that spent more on these areas had a lower proportion of study participants taking more than five medicines. At the same time, this ratio was found to be directly proportional to the expenditure recorded, as shown in Figure 1.

**Table 2:** Data collected during research Correlations between education expenditure per capita, healthcare expenditure per capita, and percentage of polypharmacy prevalence are statistically significant (p < 0.05). Reference: [26,56,58-62]

Country	GDP per capita	Government expenditure on education (% of GDP)	Education expenditure per capita	Healthcare expenditure per capita	Percentage of polypharmacy prevalence 65+ years
Sweden	$51610.00	7.641	$3943.52	$5671.39	31.00%
USA	$46195.00	5.262	$2430.78	$5048.37	36.80%
Spain	$29614.00	4.178	$1237.27	$2711.19	31.60%
Belgium	$46117.00	6.377	$2940.88	$4960.39	34.00%
Switzerland	$81994.00	4.861	$3985.73	$9666.34	11.80%
Jordan	$4330.00	2.988	$129.38	$334.04	82.70%
Poland	$15598.00	4.616	$720.00	$1014.04	33.80%
Romania	$12920.00	3.345	$432.17	$738.56	86.33%

Conclusions About LoE

In conclusion, more research on the correlation between LoE and the prevalence of polypharmacy is needed. It seems that spending more than 12 years on education may have a positive effect on medication adherence since 10-12 years of education are compulsory for everyone in many countries; thus, spending voluntarily more time on education may indicate higher intellectual capacity, resulting in better control of polypharmacy and knowledge about drugs.

The conclusions after ranking countries by LoE are unclear. The studies conducted showed that the prevalence of polypharmacy in patients aged 65+ (in Jordan, 60+) may not be correlated with the LoE in each country (PISA score), although the prevalence still seems to be slightly higher in countries with lower levels of education like Jordan.

Education is undoubtedly a crucial factor, but in summary, it is difficult to isolate one factor on overall polypharmacy levels, and more socioeconomic variables need to be considered. More caution is required for cross-country comparisons because certain educational programs cannot be easily classified and do not reveal the quality of education itself.

Undoubtedly, more studies need to be conducted on the topic of education relevance in the prevalence of polypharmacy. The role of years spent during education, expenditures on education in certain countries, and the role of age and gender are not entirely clear.

Doctor-dependent factors

Doctor-Patient Relationships

The willingness to decrease the number of medications taken daily was shown to be present among older patients in various societies [63,64]. The majority of them (78-85%) believed that all medications taken were necessary; however, 89% would like to stop one or more of their regular medications if their doctor said it was possible [63,64]. Also, doctors are advised to take deprescribing into routine clinical care [65]. Unfortunately, this is not a common practice due to a lack of inappropriate polypharmacy awareness, low self-perception of communication skills, and visit time [65]. Let us consider the selected challenges.

Appointment time: The first problem is time. The average visit length in the USA ranged from 9.7 minutes to 23.3 minutes (median: 15.7) in 1998-2000. About five minutes were spent on the most extended topic, whereas the remaining issues each received 1.1 minutes. While time spent by patients and physicians on a topic responded to multiple factors, the length of the visit overall varied little, even when the contents of visits varied widely [21]. Thus, the time for polypharmacy analysis is not optimal. A median of only five minutes was spent on even the major topic of a visit. Researchers also found that visit length was not correlated to the contents of a visit. The longer time spent on major topics seemed to have been compensated by limiting the time allocated to minor topics, therefore leaving the visit length more or less the same [21]. For instance, a patient with severe heart problems, existing diabetes, and hypercholesterolemia does not draw attention to drug numbers, methods, or routes of administration. The trust is given to the physician, who rarely initiates polypharmacy as a main or even minor visit topic due to a feeling of a lack of visit time. Prasad et al. showed that 67% of clinicians needed more time for new patients, and 53% needed additional time for follow-up appointments [66]. Time pressure was more present in internal medicine doctors than in general practitioners (GPs) (74% vs. 55%, p < 0.05), women clinicians, and chaotic workplaces. Thus, clinicians had higher levels of burnout, stress, and intent to leave. Those factors were linked to patients’ outcomes, such as satisfaction and adherence, and indirectly to polypharmacy. The doctors are forced to give up other topics during visits, like the issue of taking medications, as evidenced by the difference between the time allotted and the time needed for first and follow-up visits. The exact values are shown in Table 3.

**Table 3:** Difference between need and allotted visit time min: minutes; SD: standard deviation Table adapted from Prasad et al. [66].

	Time allotted (min)	Time needed (min)	% clinicians requiring more time than allotted
First visit	35.2 (SD = 10.4)	45.9 (SD = 10.5)	67
Follow-up visit	19.9 (SD = 3.5)	23.5 (SD = 6.0)	53

Worryingly, the Minimizing Error, Maximizing Outcome (MEMO) study in 2009 showed that time pressure for patients has constantly increased (from 53% to 67% of clinicians in 2015) [67]. It seems this trend will not be reversed in the near future.

The solution to the lack of time problem may be creating software tools and appropriate criteria. Repeatedly used throughout history, scales are the easiest way to fight polypharmacy and an essential solution [68]. Among the literature reviewed, the most frequently mentioned are “The Medication Appropriateness Index” and national lists of potentially inappropriate medication use in clinical practice like “Screening Tool to Alert Doctors to the Right Treatment,” “Screening Tool of Older Persons’ Potentially Inappropriate Prescriptions,” and “Assess, Comprehensive Geriatric Assessment, Adherence, Development, Emergence, Minimization, Interdisciplinarity, Alertness” (ACADEMIA) [69-71]. ACADEMIA acronym is an eight-step conceptual basis for improving and continuing drugs by assisting doctors in the decision-making process of whether medication should be continued or discontinued, given in a modified dose, or replaced [69]. However, given criteria or guidelines should be adjusted to the major as well as minor issues the patient is dealing with. A good example is the research group that created OncoSTRIP [72] to optimize polypharmacy in older patients with various cancers. The method includes a polypharmacy anamnesis, a brief patient assessment, a polypharmacy analysis that takes life expectancy into account, and an optimized treatment plan. After integrating it into routine care, 41% of potential drug-related problems (DRP) decreased, and DRP reduction among cancer patients was 30% [72]. Another approach to the problem is to develop software [73] to assist medical personnel. A preliminary study [74] presented a pilot computer tool created to ease the review of drug lists and alarm potentially inappropriate polypharmacy found in its database. The time reduction was at least 79.2% (from over 30 minutes per patient with paper and pencil to 6.26 minutes per patient with the used tool) [74]. In the future, universal access to similar programs might be a game changer in primary and hospital care, saving time, money, and patients' lives.

Doctor-patient communication skills: The second issue is doctor-patient communication skills. Various authors pointed out numerous mistakes made in this field. Educating patients comes to the fore. Studies for years have shown that patients with higher knowledge have better polypharmacy outcomes [15,20,73,75]. For example, the Eliminating Medications Through Patient Ownership of End Results (EMPOWER) cluster study [76] found that elders, after receiving and reading a short booklet with information like the risks of benzodiazepine products and including a self-assessment section, had a higher discontinuation rate (27% in the intervention group compared to 5% in the control group) of drug use [75,76]. Also, dose reduction occurred in an additional 11% of patients [76]. Not only doctor-to-patient education influences adherence and polypharmacy. Pure knowledge is essential, but also soft skills play a critical role in the doctor-patient relationship [73]. Undoubtedly, patients benefit from a trusted relationship with their GP. Moreover, they should be empowered to voice concerns, particularly difficult ones, and be aware of the importance of their involvement in the fight against polypharmacy. Additionally, elders with polypharmacy might gain from having a greater awareness of the reasons why older adults may require different medications, as well as the advantages of deprescribing some of their previously used drugs [75]. Thus, national medical organizations should organize such training for clinicians to improve doctor-patient relationships and encourage inter-specialty collaboration between doctors and pharmacists to provide the best possible care to patients [73].

As mentioned above, doctors should be aware of the problem and get further training to progress on the issue of polypharmacy and interdrug interactions among their patients [20]. Popularizing the use of drugs that combine multiple therapeutic substances in one easy-to-take form of medication should be the responsibility of both pharmaceutical companies and the prescribers themselves.

In conclusion, doctors may have no time or skills to respond to polypharmacy. At the same time, there are plenty of different solutions that could solve the problems presented. Everyone would benefit, from doctors and patients (their safety and QoL) to the national health care system.

Knowledge of Disease

Last but not least, knowledge of disease should be raised as a factor linked with polypharmacy. Although it was chosen to classify it as a doctor’s dependent factor, it should be remembered that knowledge of disease may be closely correlated with LoE at least in some cases. When discussing polypharmacy and related issues, the question should be raised if the patients are aware of the disease they suffer or the purposes of prescribed medications. According to current knowledge, this has a major impact on the patient’s compliance with the doctor’s recommendations and remains unchangeable over decades [19,42]. Therefore, this problem should be thoroughly analyzed, and in turn, in this part, we decided to summarize the available literature on this topic.

First of all, there are several studies analyzing directly the problem regarding the lack of knowledge about prescribed drugs among patients. A study conducted on community-dwelling older patients with polypharmacy [41] tried to assess their ability to indicate the purposes of prescribed medications. Interestingly, only 15% of the studied population were shown to know all of the indications correctly. Unsurprisingly, a negative correlation was observed between the number of drugs and knowledge. Similarly, this correlation was confirmed by studies performed by other research groups [77,78], which showed that the ability to name the indications properly decreases with the growing number of medications prescribed. Importantly, other researchers [77] accomplished an analysis of older patients in primary care units, while Peacey et al. assessed a highly specific group involving adult patients with acute psychiatric illnesses taking sleep medications [78]. Therefore, the problem was shown to be present among diverse populations. In another research focusing on a similar problem [79], the average number of drugs taken by patients with no knowledge about their treatment was almost nine, which undoubtedly meets the polypharmacy criteria. Moreover, the researchers concentrated on differences between hospitalized and ambulatory patients, and significantly more of the latter were able to indicate their medications correctly. Finally, the Italian group [64] conducted a study on older patients with polypharmacy, showing that knowledge about their medications decreased with age. This study showed that almost 90% of patients wished to reduce the number of prescribed drugs. Additionally, inappropriate medication use among patients with polypharmacy is a well-known problem [80]. Therefore, further studies should be conducted on deprescribing medications to reduce polypharmacy.

Interesting observations were held by other researchers [81], who conducted a study on patients after a stroke or transient ischemic attack (TIA). The researchers assessed the patients’ knowledge of the disease risk factors, contrary to the studies mentioned above focusing on knowledge of prescribed drugs. Interestingly, the risk factors of the stroke or TIA were known significantly better by patients with polypharmacy. However, the overall knowledge remained poor.

**Figure 2:**
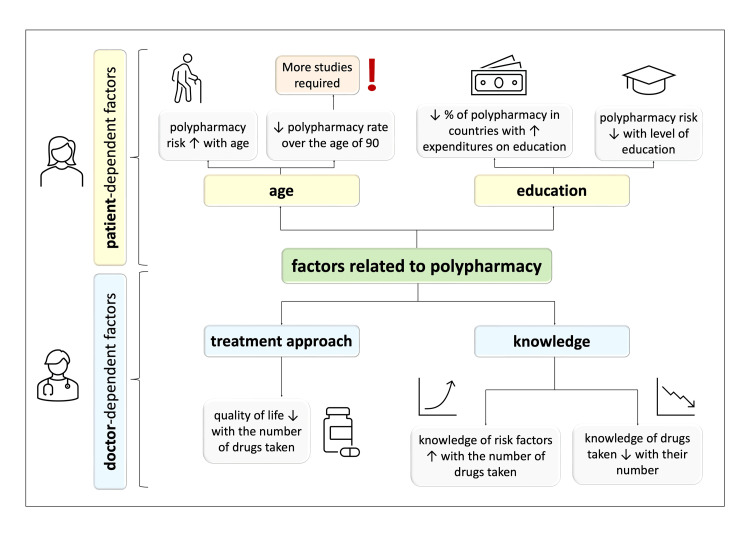
Summary of the main factors related to polypharmacy appeared in analyzed studies ↑: increase; ↓: decrease Credit: Image created by the author

Another issue to discuss, on the border of the two mentioned topics, knowledge of the disease and the doctor-patient relationship, is the need for improving doctor-patient communication regarding the disease, as well as patients’ medical education. A Denmark study [82] noticed the problem and suggested that improvement in this area may result in treatment optimization among patients with polypharmacy. However, several barriers have been indicated in patients’ better involvement in their treatment with time pressure at the top. Importantly, the researchers mentioned factors that improved the situation, for instance, providing a patient with a simple list of medications. Another research group [75] conducted a study on patients with polypharmacy who were shown not to be aware of their active role in their disease management. A need for change in this area was suggested. Therefore, diverse ideas for effective methods in improving patients’ medical education and other factors, shown in Figure 2, should be widely analyzed and may be the field for further studies.

## Conclusions

Our study underscores that polypharmacy is influenced by multiple interconnected factors. Research has identified four key factors correlated with the issue: two patient-dependent factors - age and education - and two doctor-dependent factors - general knowledge and QoL. Notably, while increased age generally correlates with higher medication use, this trend reverses after the age of 90, potentially due to better health in this age group. Additionally, while national education levels and PISA scores seem to have minimal impact on the issue, individuals with over 12 years of education tend to use fewer medications, highlighting the role of personal education in managing polypharmacy. Consistently, both higher levels of patients’ education and increased costs of education and health care incurred by countries were positively correlated with a lower number of medications prescribed. Thus, further research in this direction is needed to better understand the role played by the school system. Doctor-dependent factors, such as the patient's QoL and knowledge about their medications, are crucial in addressing polypharmacy. There is a clear link between the number of medications a patient takes and their understanding of those medications, emphasizing the importance of careful prescription practices and patient education. Interestingly, patients with polypharmacy often have greater awareness of disease risk factors and vice versa, though this should not be the sole reason for patient education. Doctors should utilize modern tools and technologies to manage the issue more effectively, as unnecessary medications are sometimes unnecessary, do not bring as much benefit, and reduce the patient's QoL.

Overall, to find the most effective strategies for treating patients with polypharmacy, researchers should closely investigate the aforementioned risk factors, paying special attention to the effects of the patient's education level, knowledge of the disease, and doctor-patient relationships. Consequently, education is necessary for GPs and other experts who deal with this obstruction on a daily basis, as well as academics around the world who have a duty to alert young students to the problem of polypharmacy during their studies. Furthermore, policymakers should consider increasing spending on education and healthcare, as this may resolve clinical and economic concerns related to the issue.
